# Bayesian melding for estimating uncertainty in national HIV prevalence estimates

**DOI:** 10.1136/sti.2008.029991

**Published:** 2008-07-22

**Authors:** L Alkema, A E Raftery, T Brown

**Affiliations:** 1 University of Washington, Center for Statistics and the Social Sciences, Seattle, Washington, USA; 2 East-West Center, Honolulu, HI, USA

## Abstract

**Objective:**

To construct confidence intervals for HIV prevalence in countries with generalised epidemics.

**Methods:**

In the Bayesian melding approach, a sample of country-specific epidemic curves describing HIV prevalence over time is derived based on time series of antenatal clinic prevalence data and general information on the parameters that describe the HIV epidemic. The prevalence trends at antenatal clinics are calibrated to population-based HIV prevalence estimates from national surveys. For countries without population based estimates, a general calibration method is developed. Based on the sample of calibrated epidemic curves, we derive annual 95% confidence intervals for HIV prevalence. The curve that best represents the data at antenatal clinics and population-based surveys, as well as general information about the epidemic, is chosen to represent the best estimates and predictions.

**Results:**

We present results for urban areas in Haiti and Namibia to illustrate the estimates and confidence intervals that are derived with the methodology.

In 2003, UNAIDS included information on uncertainty in its estimates and projections by calculating and presenting plausibility bounds. These bounds were derived by combining the results of a bootstrap method with expert opinion regarding the range of possible epidemic curves, as explained by Grassly *et al*.^1^ The method was further developed by Morgan *et al*.^2^ The results of the method, the plausibility bounds, are not formal statistical confidence intervals. Alkema *et al*^3^ proposed using Bayesian melding for uncertainty assessment in the model in the Estimation and Projection Package (EPP), the package used by UNAIDS and country officials for prevalence estimation and prediction. Bayesian melding provides a way of including expert opinion while still giving formal statistical confidence intervals. It is a method for assessing uncertainty about the inputs and outputs of a deterministic model, such as the EPP model. Bayesian melding was first developed to estimate the rate of increase of whale populations,^4^ ^5^ and was successfully applied to policymaking in that context. When applying this approach to the EPP model, it gives a “best” trajectory of HIV prevalence over time, as well as the uncertainty in the estimates and projections in the form of a set of possible epidemic curves. These curves reflect the uncertainty about the past and future and are based on the (imperfect) information that is available about the model’s inputs and outputs.

In 2006 the UNAIDS Reference Group on Estimates, Modelling and Projections recommended the use of Bayesian melding for uncertainty assessment in EPP.^6^ The application of Bayesian melding to the EPP model is discussed in detail by Alkema *et al*.^3^ Brown *et al*^7^ discuss the implementation details. In this paper we summarise the approach in a less technical way in the Methods section and discuss the recent changes based on recommendations by the UNAIDS reference group: the prior distributions on the input parameters and the calibration of prevalence curves based on ANC prevalence to the outcomes of population-based surveys. We give results for Haiti and Namibia and end with a discussion of the method.

## METHODS

### Bayesian melding for the Estimation and Projection Package

In Bayesian melding, “melding” refers to combining (melding) information about the inputs and outputs of a deterministic model, such as the model in EPP for generalised epidemics.^8^ In the EPP model, the population of 15–49-year-olds is divided into three groups, a not-at-risk group, an at-risk group and an infected group. Three differential equations describe the changes in those groups over time, and thus in prevalence over time. Four parameters determine the shape of the epidemic curve: *r*, which is the growth rate of the epidemic, *f*_0_, the fraction of population initially at risk, *t*_0_ the start year of the epidemic (in which a fraction of the population that is initially at risk gets infected) and *φ*, a parameter that modulates recruitment to the at-risk population. Information about the inputs and outputs in the EPP model is given by expert knowledge about the input parameters and upper bounds on prevalence in certain years, and prevalence data.

“Bayesian” refers to Bayesian inference, which starts by quantifying prior beliefs (expert knowledge) about the true value of a quantity of interest. For example, in HIV estimation using EPP, there could be a consensus that the generalised epidemic started no sooner than 1970 and no later than 1990. The upper and lower bounds on the start year of the epidemic serve as boundaries on any projections that would then be developed. In Bayesian inference, prior beliefs are represented by probability distributions—for example, the start year of the epidemic could have a uniform distribution on the interval between the upper and lower bounds. Likewise, prior distributions can be determined for the other three parameters in the epidemic curve fit, as well as for the output of the EPP model, prevalence over time. For example, there could be a consensus that prevalence in 1980 was no higher than 5%.

After specifying the prior distributions, they are used to generate a set of possible epidemic curves (curves that are based on the EPP parameters and satisfy the constraints imposed on prevalence). The prior distributions are then updated based on the observed outcomes of comparing these curves with the observed data. For HIV prevalence estimates with the EPP model, HIV prevalence is observed at antenatal clinics, and for many countries population surveys are available as well. Data and information on measurement errors are used to calculate a measure of the so-called “likelihood”: an epidemic curve which is similar to the level and trend in observed prevalence has a high likelihood of representing true prevalence.

Combining prior distributions with likelihood (updating prior beliefs) gives the “posterior” distribution of the quantity of interest. Melding the prior distributions on inputs and output with the likelihood on output gives posterior distributions on inputs as well as outputs—for example, the posterior distribution of past and future prevalence.

The Bayesian melding approach for EPP produces a sample from the posterior distributions on inputs and outputs. All the information that is needed can be derived from this sample. We will discuss how the best prediction is calculated from the posterior sample of prevalence curves, as well as how the confidence intervals (or so-called “uncertainty bounds”) are constructed.

### Constructing a sample of prevalence curves

We now describe how to get a sample of prevalence curves based on data from antenatal clinics (ANC). For many countries, these datasets are the main source of the times series of prevalence data that are needed to estimate the trend in HIV prevalence. Nationally representative population surveys, including the demographic and health surveys (DHS) and AIDS indicator surveys (AIS), are available for an increasing number of countries.^9^ These data are used to calibrate the ANC prevalence, as discussed below.

The sample from the posterior distribution of HIV prevalence curves is drawn using the sampling importance resample (SIR) algorithm^10^ ^11^ as follows:

- Sample a large number of different combinations of the input parameters *r*, *f*_0_, *t*_0_ and *φ* from their prior distributions. To draw one value from the joint prior distribution of the four input parameters, we proceed as follows. For each of the four parameters, we randomly sample one value from that parameter’s prior distribution. Because the four parameters are statistically independent in the prior distribution, this is a draw from the joint prior distribution.
- For each combination of the input parameters, run the EPP model to produce the corresponding epidemic curve.
- Each curve is compared to HIV prevalence data from antenatal clinics and assigned a weight based on the likelihood of the curve (how well it fits the data) and the previous assumptions on prevalence. The calculation of likelihoods and weights is discussed in detail by Alkema *et al*.^3^ For example, if the epidemic curve, as generated by the various combinations of input parameters, is very different from the observed rates, that curve will get a low or zero weight. If the curve resembles the data reasonably well, it will get a high weight. If the curve falls outside any user established previous limitations on prevalence (for example, if the expert user specified prevalence in 1999 is less than 5% and the curve being compared has a prevalence of 9%), its weight is set to zero.
- The epidemic curves as well as their input parameters get resampled, based on these weights. The probability of resampling is proportional to the weight that has been assigned to that curve.

The result is a sample from the posterior distribution of prevalence, in the form of a set of prevalence curves. The number of sets of input parameters that are sampled in step 1 should be large enough such that they cover the input space and a reasonable number of unique curves get selected in the last step—for example, at least 100 curves. We found 200 000 sets of inputs to work well for most countries to cover the input space. A much smaller number is needed in the resample step^5^—for example, 3000 curves, to get a sample from the posterior. These sample sizes are rough guidelines, and depend on the dataset that is being used to analyse HIV prevalence over time.

### Prior distributions

Prior distributions on the four input parameters to the EPP model are specified using expert knowledge. They should be broad enough so as not to exclude values that are supported by the data (that is, that have high likelihood) unless there is strong knowledge that excluding some values is appropriate. In the EPP 2007 computer program, default prior distributions are given for each of the four input parameters. The prior distribution for the fraction initially at risk, *f*_0_, is uniform between 0 and 1, and the start year of the epidemic, *t*_0_, is discrete uniform on {1970, …, 1990}. If the default limits on these distributions are too constraining, users can change them to ones that are appropriate for their own country’s situation—for example, in many Asian countries, a more reasonable range of start years might be 1980 to 2000.

**Figure 1:**
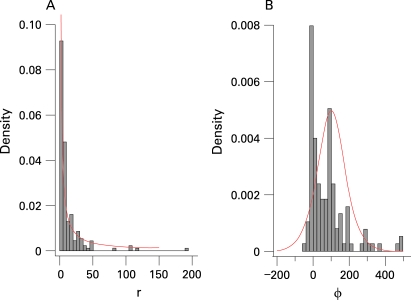
Histogram of UNAIDS 2005 estimates for (A) *r* with density of exp(U[log(0.5), log(150)]) in red and (B) *φ* with density of logistic(100,50) in red.

Values of the rate of increase, *r*, as used in the country projections by UNAIDS in 2005, are shown in figure 1A. Based on this, the default prior distribution for *r* in EPP 2007 was set to be uniform on the log scale between 0.5 and 150; its density function is shown in the figure.

Figure 1B shows the estimates of the behavioral response *φ* from 2005 (excluding a few very large values, for easier visualisation). The default prior distribution chosen for the behavioural response *φ* is drawn in the same figure and is given by:

*φ∼Logistic*(100,50) (1)

such that *φ* ranges roughly between −50 and 250, centred around 100. This is broad enough to cover the range of the previous estimates, and also reflects the expectation that the behavioural response parameter is unlikely to be highly negative.

EPP also allows the user to specify upper or lower bounds on prevalence in certain years—for example, restricting prevalence in 1980 to be smaller than 1% to exclude unrealistic epidemic curves that peak around that year at high prevalence levels.

### Calibrating ANC prevalence curves

ANC data are used for estimating the trend in HIV prevalence as for many countries these datasets are the main source of times series of prevalence data. Because of the difference between ANC prevalence and population prevalence as measured by population-based surveys as discussed by Garcia-Calleja *et al*^9^ and Gouws,^12^ calibration of the prevalence curves based on, and thus representing, ANC prevalence is needed. This section discusses the calibration of HIV prevalence for countries in which one or more population surveys have been conducted, and the calibration method for countries without population surveys, based on observed differences between ANC and population prevalence in other countries.

### Countries with population-based survey(s)

For countries with one population estimate, a calibration constant is calculated such that median posterior prevalence in the year of the survey as given by the Bayesian melding procedure based on ANC data will be rescaled at the population estimate. As in the paper by Alkema *et al*,^3^ differences in prevalence levels will be modelled on the probit scale. This scale is chosen such that differences between prevalence levels do not depend on the level itself (the probit transformation approximately stabilises the variance). The calibration constant, the difference between population prevalence and ANC prevalence, is constant over time on the probit scale, such that the influence of the calibration constant decreases for lower prevalence. We define probit transformed observed HIV prevalence as measured in population survey in year *t* as:

*W_pop,t_* = *Φ^−1^(x_pop,t_*) (2)

where Φ(·) is the standard normal cumulative distribution function and


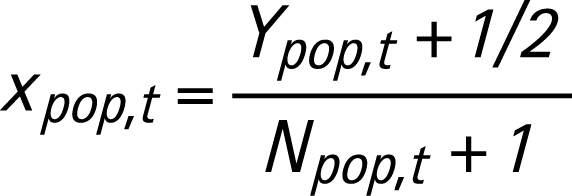


In equation 3, *Y_pop,t_* is the number of HIV-positives in the DHS sample in year *t* and *N_pop,t_* is the sample size of the population survey. The constants 1/2 and 1 in equation 3 are included to avoid problems with zeros. We denote the probit transformed curves representing ANC prevalence by ***V****_ANC_* and the transformed median estimate in year *t* by *V*∧*_ANC,t_*. The calibration constant for the DHS survey in year *t* is denoted by *c_t_* and given by:

c_t_ = W_pop,t_– V∧_ANC,t_ (4)

For countries with one survey, the final calibration constant is *c = c*_t_, and is added to the trajectories of antenatal clinic prevalence to get the calibrated population prevalence:


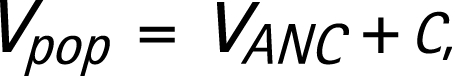



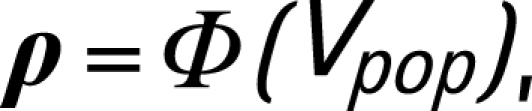


where ***V**_pop_* is a sampled calibrated epidemic curve on the probit scale, and ***ρ*** is a sampled trajectory of HIV prevalence representative for the overall population. Note that calibration is done separately for urban and rural areas.

In order to take into account the uncertainty in the population estimate the calibrated curves need to be resampled, with weights proportional to the population survey likelihood, to incorporate the likelihood of the population estimates in the posterior sample of prevalence curves. As for the antenatal clinic data given by Alkema *et al*,^3^ we derive the likelihood for DHS data by assuming that the sampling error is normally distributed on the probit scale:

*V_pop,t_*|*W_pop,t_*∼*N*(*W_pop,t_*, *Var*(*W_pop,t_*)) (7)

The variance of the population estimate is based on the binomial variance in the sample, multiplied by the design effect to take into account the cluster sampling procedure. The design effect for a cluster design is the ratio of the variance for that design to the variance calculated from a simple random sample of the same size. In EPP 2007 the default value for the design effect is 2, according to UNAIDS guidelines for measuring prevalence in population-based surveys.^13^ With design effect *δ*, the variance of the population estimate on the probit scale is approximately equal to:


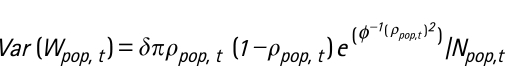


where *ρ_pop,t_* is the true population prevalence rate. Estimating *ρ_pop,t_* by *x_pop,t_* gives an estimate of the variance on the probit scale. The likelihood of the population data given a calibrated epidemic curve can now be determined using equation 7. An example of the calibration is given in the Results section for Haiti.

For countries with more than one survey, the calibration constant *c* is given by the mean of each of the survey-specific calibration constants. Using weights in the resample step that are proportional to the product of the likelihoods of the population surveys results in higher weights for curves that best resemble the trend as given by the population estimates, thus confidence intervals that incorporate the trends as given by the population estimates.

### Countries without a population-based survey

EPP allows ANC prevalence estimates for countries without population-based estimates to be adjusted based on the biases revealed by population surveys in other countries. We assume that there is an overall bias that gives the difference between ANC and population surveys. This bias is unknown, because of the measurement errors in both the ANC and the population estimates. The mean bias and its standard error are estimated from the data from countries with population surveys, separately for urban and rural areas. These estimates are used to adjust ANC prevalence in countries without population prevalence estimates.

Observed differences between ANC prevalence and DHS estimates are used to calculate calibration constants on the probit scale. The distribution of the calibration constant is based on the observed ANC bias in 17 countries in sub-Saharan Africa (Burkina Faso, Burundi, Cameroon, Chad, Ghana, Guinea, Ethiopia, Kenya, Lesotho, Malawi, Mali, Niger, Rwanda, Sierra Leone, Tanzania, Uganda and Zambia). The adjustment for rural areas is bigger than for urban areas, so we derive the distribution of the calibration constant for those two subpopulations separately. On the probit scale the mean calibration constant (difference between ANC and DHS prevalence) for urban areas is 0.11 with standard error 0.04, for rural areas 0.17 with standard error 0.05.

To construct calibrated prevalence curves that are representative for the overall population in urban areas, a calibration constant *c_urban_* is sampled and added to ***V****_ANC_*:

*c_urban_*∼*N(0.11,0.04^2^)* (9)


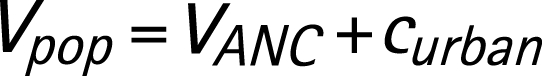


***ρ*** = *Φ(V_pop_*)(11)

where ***V****_ANC_* is a sampled trajectory of ANC prevalence on the probit scale, ***V****_pop_* is the corresponding calibrated epidemic curve on the probit scale and ***ρ*** is the resulting sampled trajectory of HIV prevalence for the overall population, in this case the population living in urban areas. The adjustment for rural epidemics is done similarly, with:

*c_rural_*∼*N(0.17,0.05^2^)* (12)

Table 1 summarises the effect of the calibration when ANC prevalence is 5%, 10%, 15% and 20%. For example, if ANC prevalence is 15% in urban areas, urban population prevalence is estimated as 12.6% (95% confidence interval (CI) 11.2% to 14.1%). The downward adjustment is larger for rural prevalence—for example, if ANC prevalence is 15%, the adjusted prevalence in rural areas is 11.3% (95% CI 9.6% to 13.3%).

**Table 1:** Adjustments of ANC prevalence for urban and rural areas with 95% confidence intervals for the adjustment

ANC prevalence (%)		Urban adjusted prevalence (%)		Rural adjusted prevalence (%)
5		4.0 (3.4 to 4.6)		3.5 (2.8 to 4.3)
10		8.2 (7.2 to 9.3)		7.3 (6.0 to 8.7)
15		12.6 (11.2 to 14.1)		11.3 (9.6 to 13.3)
20		17.1 (15.4 to 18.9)		15.5 (13.3 to 17.9)

**Figure 2:**
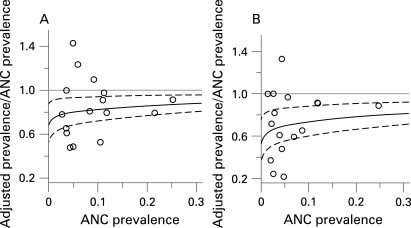
Ratio of adjusted antenatal clinics (ANC) prevalence to ANC prevalence for countries without a population survey for (A) urban and (B) rural areas. The grey line is the identity line representing no adjustment. The solid black lines are the adjustments of ANC prevalence based on the observed mean adjustments (on the probit scale) in other countries. The broken lines give the 95% CI for the ratio of estimated prevalence to ANC prevalence after adjustment. The dots are the observed outcomes of the ratio of DHS surveys to estimated ANC prevalence.

Figure 2 shows the ratio of adjusted (calibrated) prevalence to ANC prevalence for ANC prevalence ranging from 0 to 30%. The observed ratios of DHS surveys to ANC prevalence are shown in the same figure. The results of this calibration method are illustrated for urban areas in Namibia in the Results section.

### Best estimate and confidence intervals

Based on the (calibrated) sample of curves from the posterior distribution of HIV prevalence curves, a 95% confidence interval for prevalence in a given year is given by the lower 2.5th and the upper 97.5th percentiles of the prevalences for that year within the sample. The “best” estimates are given by the trajectory that is most likely to represent prevalence over time, given prior distributions and data. This maximum a posteriori (MAP) trajectory is the one with the highest posterior density, proportional to the product of the prior distributions on the inputs of the curve, the prior distributions on outputs and the likelihood of the data. For countries without a population estimate, the best calibrated prediction is given by the MAP curve as derived for ANC data, with the mean calibration constant added to it. For countries with one or more population estimates, the maximum a posteriori curve is given by the calibrated curve with the maximum product of input priors, ANC likelihood and population-based likelihood.

Epidemics are fitted separately for urban and rural areas, giving so-called “sub-epidemics” within one country. For deriving nationally representative estimates and projections, sub-epidemics are combined by sampling trajectories from each sub-epidemic, weighting each trajectory by population size in each year and adding them up. Confidence intervals are then constructed based on the nationally representative sample of trajectories. Best estimates for national prevalence are given by a weighted average of the maximum a posteriori estimates of the sub-epidemics, with weights given by population size.

## RESULTS

In this section we will present the results of the Bayesian melding procedure for urban areas in Haiti and Namibia. Note that the estimates and predictions as derived here are illustrative and are not the official estimates, as our analysis might not include all the information available.

**Figure 3:**
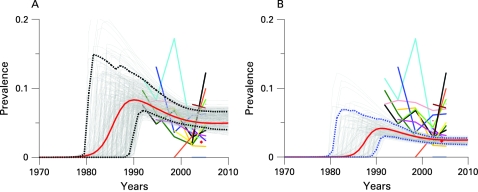
Bayesian melding for estimating and projecting HIV prevalence (on the Y-axis) in urban areas of Haiti (A) based on antenatal clinic data only, and (B) based on antenatal clinic data and the 2006 DHS survey. The coloured lines with symbols show the raw antenatal clinic prevalence rates and the red diamond shows the DHS estimate. The light grey curves are a sample from the posterior distribution of the prevalence curve, and the red curve is the posterior mode. The broken lines show the year-specific 95% confidence intervals for prevalence.

Adult HIV prevalence in the Caribbean is estimated at 1.0% (95% CI 0.9% to 1.2%) in 2007.^14^ Prevalence in this region is highest in the Dominican Republic and Haiti. In figure 3A, HIV prevalence data for urban areas in Haiti are given by the coloured lines for 17 antenatal clinics (data taken from Gaillard *et al*^15^). Sample sizes for each clinic range from 14 to 393 women, and observed prevalence in 2006–7 ranges from 0% to 12.2%. The light grey curves in the plot are a sample from the posterior distribution of the ANC prevalence curves, based on the default priors on the input parameters, and restricting prevalence in 1980 to be smaller than 1%. The red curve gives the best estimates (MAP curve) and the broken lines show the year-specific 95% confidence and prediction intervals. Based on antenatal clinic data, the posterior median of prevalence is 5.3% for 2007 (95% CI 4.3% to 6.7%).

In 2005–6, DHS carried out a population-based survey in Haiti. For urban areas, HIV prevalence was estimated to be 2.3% (with sample size 4382, design effect set to 2). In figure 3A and 3B the DHS estimate is shown by the red diamond. In figure 3B, this estimate is used to calibrate the trajectories as given by the ANC data. In this plot, each of the light grey curves represents calibrated prevalence over time, with 95% confidence intervals (broken lines) and best estimate as given by the MAP curve (red). The calibration based on the DHS estimate gives a significant reduction in estimated prevalence level and uncertainty. Based on ANC data and the DHS estimate, the posterior median of prevalence is 2.3% for 2007 (95% CI 1.9% to 2.8%).

### Namibia

**Figure 4:**
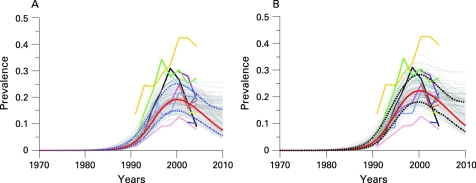
Bayesian melding for estimating and projecting HIV prevalence (on the Y-axis) in urban areas of Namibia (A) based on antenatal clinic data only, and (B) calibrated to represent prevalence among the overall urban population. The coloured lines with symbols show the raw antenatal clinic prevalence. The light grey curves are a sample from the posterior distribution of the prevalence curve, and the red curve is the posterior mode. The broken lines show the year-specific 95% confidence intervals for prevalence.

Namibia is one of the countries in southern Africa with high HIV prevalence levels. Figure 4 shows data from 12 antenatal clinics in urban areas (Windhoek Central Hospital, Grootfontein, Oshakati, Katutura, Rundu, Otjiwarongo, Tsumeb, Rehoboth, Gobabis, Mariental, Katima Mulilo and Walvisbay, data taken from the epidemiological fact sheets on HIV/AIDS and sexually transmitted infections 2006 at http://www.who.int/globalatlas/predefinedReports/EFS2006/index.asp and Ministry of Health and Social Services Namibia^16^). Based on the antenatal clinic data, median prevalence in 2007 is estimated at 16.9% (95% CI 13.2% to 22.1%).

Namibia has no population survey to calibrate the ANC prevalence curves. We use the general calibration method as described in the previous section. The results are shown in figure 4B. After calibrating the ANC curves, median prevalence in 2007 is estimated at 14.6% (95% CI 10.8% to 19.7%). Note the uncertainty in past estimates in HIV prevalence: the widths of the confidence intervals are approximately 10% from 1999 until 2006.

## DISCUSSION

In this article we have described the Bayesian melding approach to assessing uncertainty in HIV prevalence estimates as given by the model in the Estimation and Projection Package. Using the Bayesian melding procedure, prevalence over time is described by a sample of epidemic trajectories, which are chosen based on all available information about input parameters of the model and prevalence data. Uncertainty about prevalence is given by year-specific 95% confidence intervals.

The Bayesian melding approach allows users of EPP to get a better insight into HIV prevalence trends, specifically in the range of epidemic curves describing the situation in their area of interest and the uncertainty bounds associated with the best estimates. Compared to the previous approach for uncertainty assessment in EPP,^2^ this approach produces statistical confidence intervals and allows for incorporating expert information on model input and prevalence outcomes.

The number of curves needed to get an appropriate sample of the the posterior distribution of prevalence curves in the sampling importance resampling method depends on the dataset that is being analysed. More curves will give a more accurate result. The optimal number of curves to generate and resample depends on the pay-off of longer computations versus increased accuracy. In this paper we give some rough guidelines for sample sizes. In future work we will explore more efficient sampling algorithms and specify stopping rules.

The trend in prevalence over time is derived from what has been observed at antenatal clinics and calibrated to represent prevalence in the overall population based on population-based surveys. In the current calibration of HIV prevalence curves to population-based estimates, the latter estimates are taken to be unbiased estimates of population prevalence. Calibrating prevalence in urban areas in Haiti based on the DHS estimate of population prevalence, resulted in a lower estimate and a much narrower confidence interval for 2007.

The general calibration method for countries without population-based estimates allows for adjusting biases in antenatal clinic data compared to population estimates. This calibration method gives the overall adjustment, based on what has been observed in several countries. Using this general calibration method changes the estimates of HIV prevalence, but has less impact on the width of the confidence intervals, as illustrated for Namibia. With more population-based surveys becoming available, fewer countries will need to use the general calibration method, and country-specific calibrations can be carried out to get more accurate estimates and predictions of HIV prevalence.
